# Malignant Transformation of a Supratentorial Neurenteric Cyst: A Case Report and Literature Review

**DOI:** 10.7759/cureus.85781

**Published:** 2025-06-11

**Authors:** Nura Alanazi, Ahmed Gamal Sayed, Maher Hassounah, Mohammad Dababo

**Affiliations:** 1 Department of Pathology and Laboratory Medicine, King Saud University Medical City, Riyadh, SAU; 2 College of Medicine, Alfaisal University College of Medicine, Riyadh, SAU; 3 Neurosurgery, King Faisal Specialist Hospital and Research Centre, Riyadh, SAU; 4 Anatomic Pathology, King Faisal Specialist Hospital and Research Centre, Riyadh, SAU

**Keywords:** congintal lesion, intracranial neurenteric cyst, malignant transformation, neurenteric cyst, supratentorial

## Abstract

Neurenteric cysts (NECs) are congenital lesions of endodermal origin that are uncommon, and malignant transformation is extremely rare. They are predominantly found in the spinal subdural space. We report a case of a 50-year-old female with a known history of hypothyroidism, presenting with a nine-month history of severe occipital headache, neck pain, and rigidity, accompanied by a notable unintentional weight loss of 20 kg. Neurological examination was unremarkable. Imaging studies revealed a complex multilobulated cystic lesion involving the velum interpositum and the trigone of the left lateral ventricle. Diffuse craniocervical spinal cord leptomeningeal enhancement was identified. Lumbar puncture confirmed rare, atypical epithelial cells. Gross total resection of the tumor was done through a parietal craniotomy. Histopathological examination revealed transitional features of a neurenteric cyst from benign to malignant, supported by morphological and immunohistochemical findings. Neurenteric cysts, though rare in supratentorial or infratentorial locations, should be included in the differential diagnosis of intracranial cysts. The presence of benign epithelium and dysplastic epithelium with malignant features, together with a high proliferation index, indicates malignant transformation of the neurenteric cyst. To the best of our knowledge, this is the fourth case reported so far that is distinct regarding location, with the velum interpositum and the trigone of the left lateral ventricle rarely being reported sites.

## Introduction

Rare congenital cystic lesions of suspected endodermal origin that occasionally occur in the craniospinal axis are known as neurenteric cysts. They have basement membranes that mimic those of the intestinal or respiratory tracts and cyst walls covered with simple or pseudostratified, ciliated or non-ciliated, cuboidal or columnar epithelium [1]. In 1928, the first description of an intracranial neurenteric cyst was published. The posterior cranial fossa is the most typical location [2,3]. Supratentorial is a very rare location for the presence of NECs. Our case is the fourth one, along with the other three present in the literature, where the location of the neuroenteric cyst (NEC) is supratentorial [4-6]. The total number of cases of NEC with malignant transformation, including ours, despite location, is 10 [4,5,7-13]. The main treatment for NEC is surgery, and it is advised to completely remove the lesion while carefully protecting the surrounding structures [14]. Following surgical procedures, the prognosis is generally satisfactory [14-16]. But occasionally, the NEC remnants that result from subtotal or partial excision brought on by adhesion between the NECs and adjacent structures might grow into tumor recurrences [14]. 

## Case presentation

A 50-year-old female, with a known case of hypothyroidism, presented with a nine-month history of progressively severe occipital headaches associated with neck pain and rigidity. Remarkably, she reported an unintentional weight loss of 20 kg over the preceding six months. Denying any fever, nausea, vomiting, seizures, or focal deficits, she did reveal a positive family history of migraine.

On examination, the patient was cachectic, conscious, alert, and oriented to time, place, and person. There was mild neck rigidity, no cranial nerve palsies, and no sensory or motor deficits. Cardiovascular and respiratory system examinations were unremarkable. 

**Figure 1:**
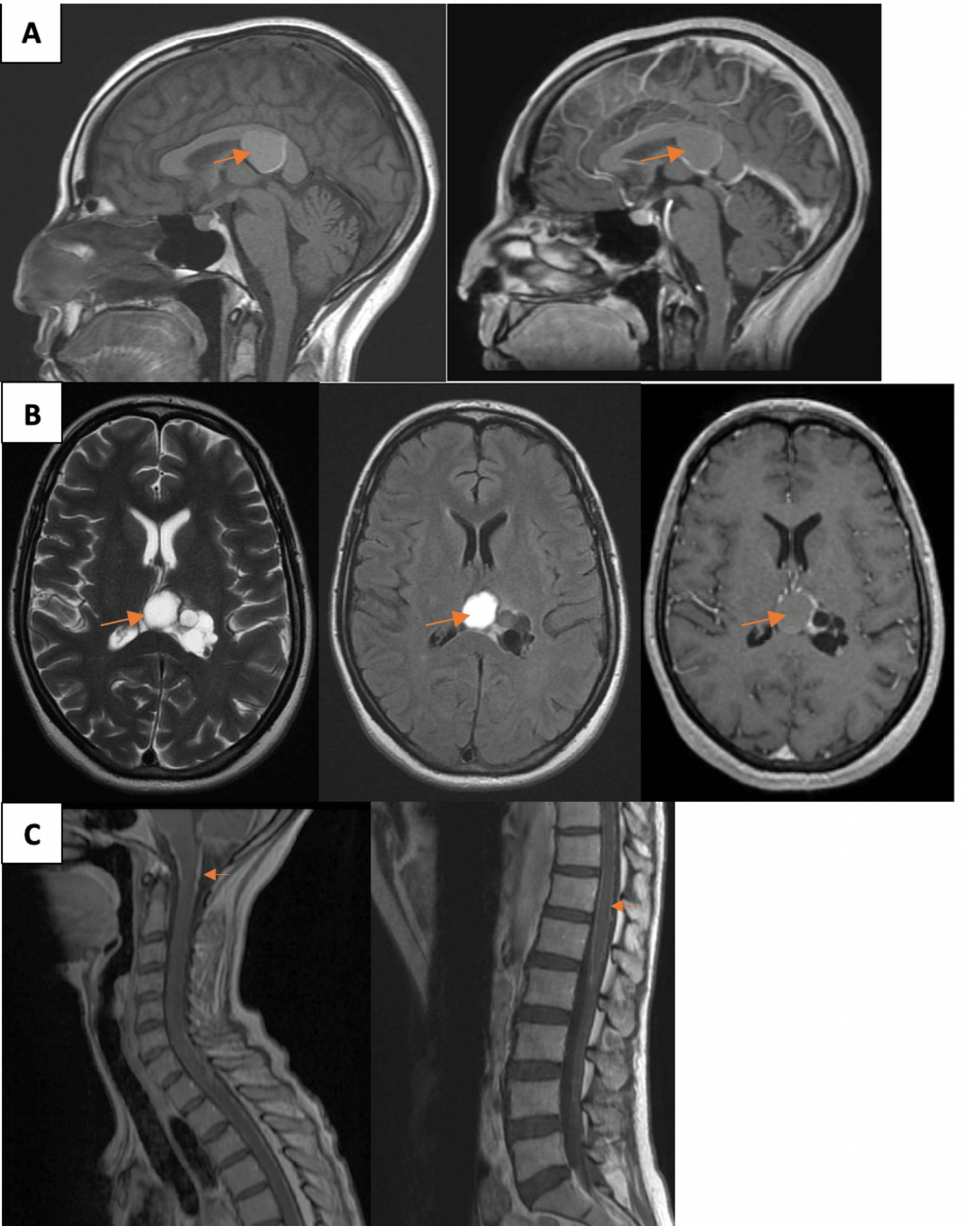
Pre-operative MRI scans of the patient. (A) Sagittal T1-weighted MRI brain without and with Gd-enhancement. (B) Axial T2-weighted, FLAIR, and T1-weighted MRI of the brain with Gd-enhancement exhibiting a complex multilobulated predominantly cystic lesion involving the velum interpositum and the trigone of the left lateral ventricle. (C) Sagittal T1-weighted MRI spine with Gd-enhancement exhibiting diffuse leptomeningeal enhancement. (MRI, magnetic resonance imaging; Gd, gadolinium).

The initial brain MRI showed a 37 x 19 mm complex multilobulated predominantly cystic lesion involving the velum interpositum and the trigone of the left lateral ventricle (Figures 1A, 1B), without evidence of hydrocephalus. 

The impression of the initial brain MRI was that the cystic lesion was likely of a benign nature. However, because of the loss of weight and to rule out the possibility of brain metastasis, a CT of the chest, abdomen, and pelvis was performed. It showed gastric polyps. Gastroscopic biopsy revealed mild nonspecific chronic inactive gastritis. MRI of the brain four months after the initial MRI showed minimal increase in the cystic lesion and leptomeningeal spread around the medulla and upper cervical spinal cord. MRI of the whole spine showed drop metastasis at the conus medullaris and cauda equina (Figure 1C). 

**Figure 2:**
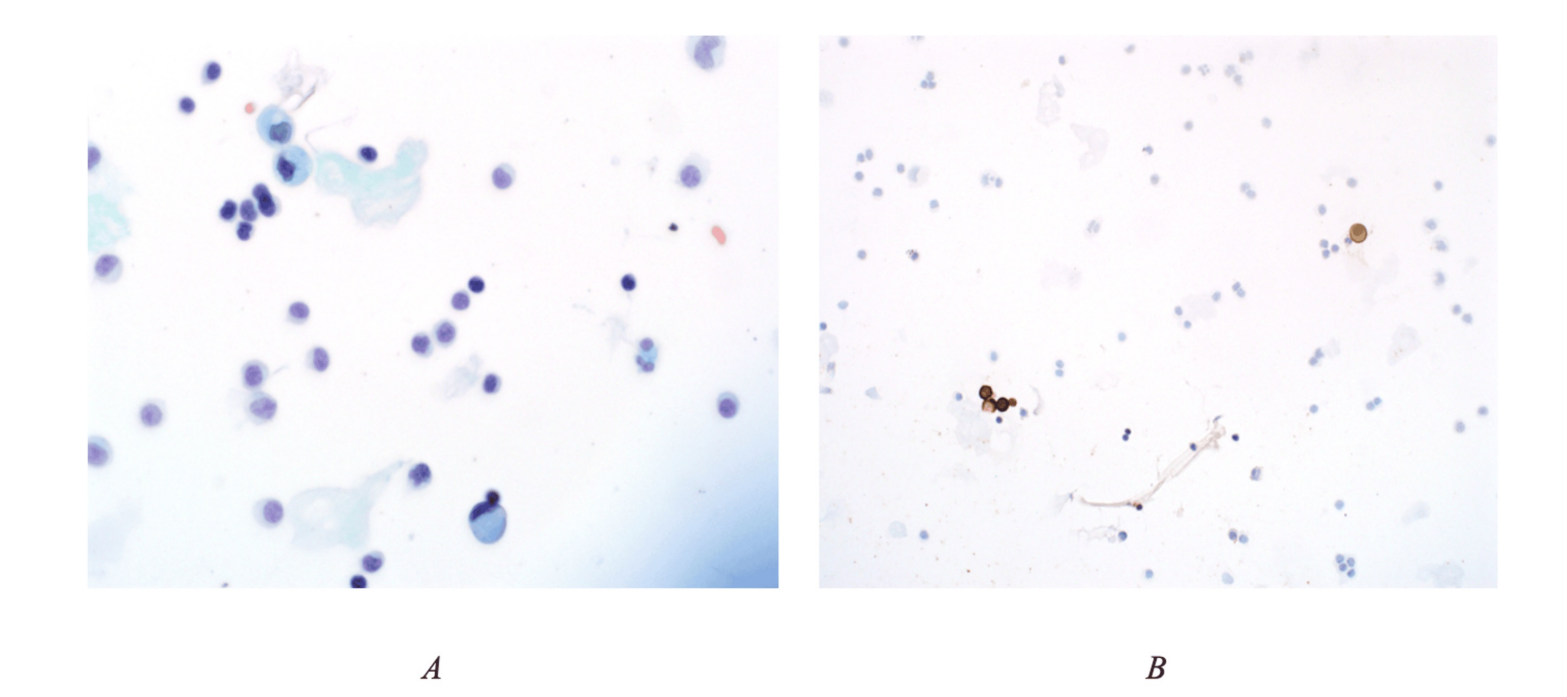
Cytological analysis of cerebrospinal fluid (CSF) showed singly dispersed, atypical epithelioid cells with irregular nuclear membranes and vacuolated cytoplasm (A. Papanicolaou stain, B. The immunohistochemical staining revealed that the atypical epithelioid cells expressed AE1\AE3.

Lumbar puncture confirmed rare, atypical epithelial cells (Figure 2).

The patient underwent left parietal craniotomy and trans-superior parietal lobule approach. A gross total resection of the cystic lesion was achieved. An early postoperative MRI of the brain confirmed the gross total resection. Recovery was uneventful.

**Figure 3:**
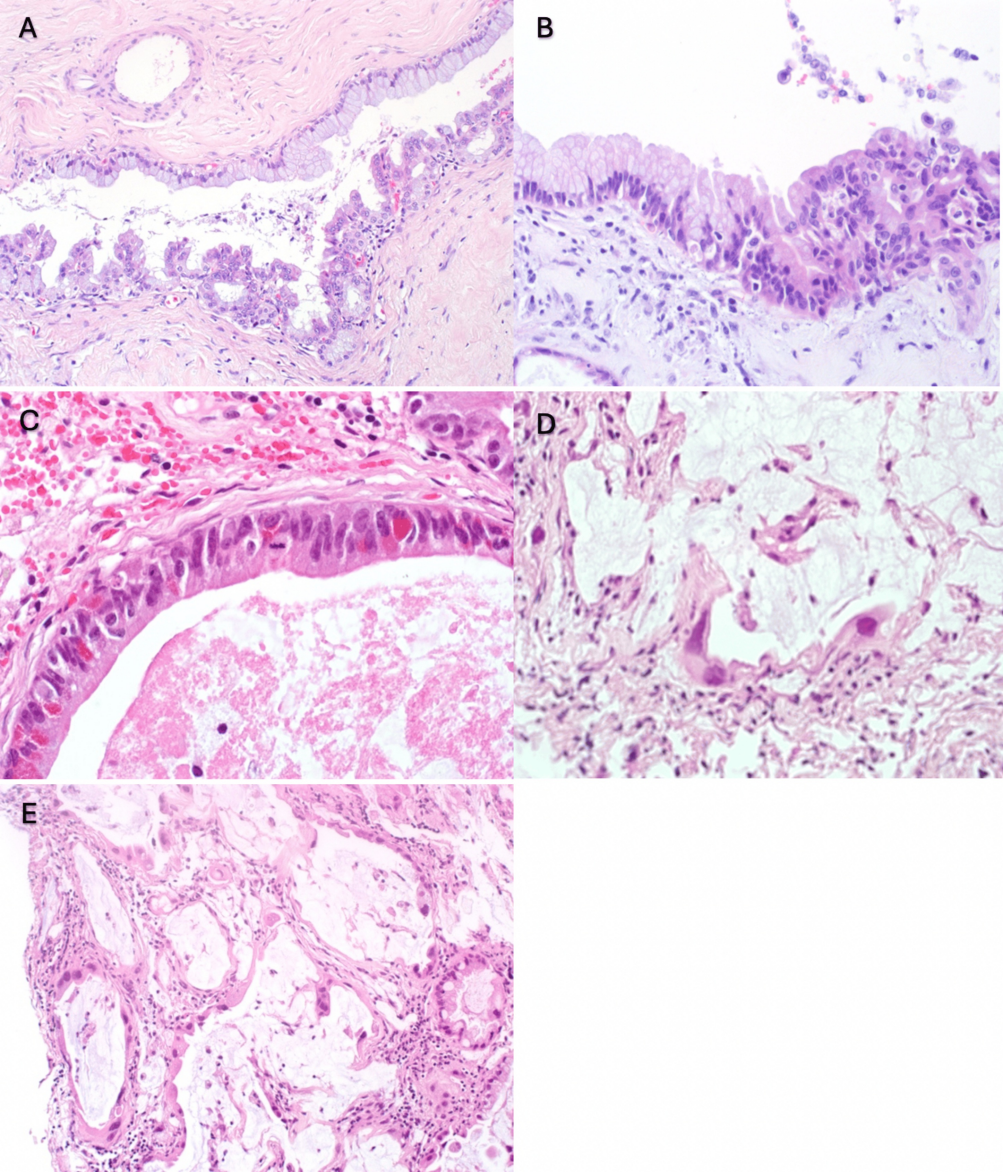
Histopathological features of the specimen obtained in the patient's craniotomy.

**Figure 4:**
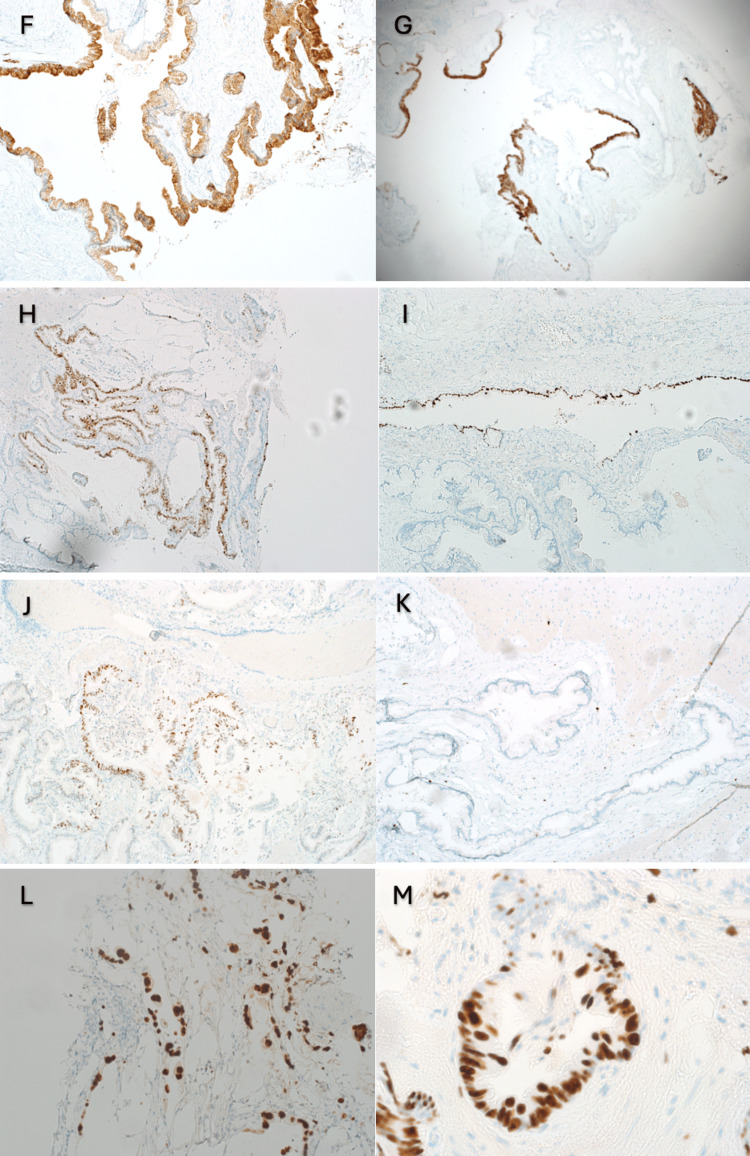
Immunohistochemical stains of the specimen.

The histological examination of the specimen revealed that the cyst wall was lined by a single layer of bland mucin-secreting columnar epithelium with features of typical NC, opposite to the area where the cyst was lined by a dysplastic epithelium with malignant features, including enlarged hyperchromatic nuclei with high nuclear-to-cytoplasmic ratios (Figure 3A). An area in which transition features of the neurenteric cyst from benign, very bland, mucinous epithelium (left) and dysplastic epithelium (right) were identified (Figure 3B). Mitotic figures were observed in dysplastic epithelium (Figure 3C). Dissecting mucin with malignant cells was also observed (Figures 3D, 3E). The immunohistological staining revealed that the cells expressed cytokeratin 7 (Figure 4F), cytokeratin 20 (Figure 4G), CDX2 (Figure 4H), and TTF1 (Figure 4I). Staining for the p53 mutation was positive in the epithelium with malignant features (Figure 4J). The MIB-1 labeling index was almost 0% in benign areas (Figure 4K) in contrast to the high percentage in areas with dissecting mucin with malignant cells (Figure 4L, 4M).

Fractionated craniospinal irradiation to a total dose of 50 Gy was completed. She developed hydrocephalus, and a ventriculoperitoneal shunt was inserted. Follow-up cranial and spinal MRI revealed increased leptomeningeal dissemination. Her level of consciousness was deteriorating, and she was fed via a nasogastric tube. She died 11 months after the craniotomy.

## Discussion

It is still unknown what exactly caused the transition and the initial NECs. According to Mittal et al., aberrant endodermal cell migration that takes place dorsally through the primitive neurenteric canal and into the ectoderm causes NECs [17]. The gradually declining incidence of intraspinal, posterior fossa, and supratentorial NECs can be explained by this approach. According to this theory, the ectopic endodermal cells that spread out the most might have had a higher chance of becoming cancerous or developing dysplasia. This theory might account for the difference in location [17]. According to other research, tissue may be more susceptible to malignant transformation if there is persistent inflammation, most likely brought on by recurrent cyst rupture or subtotal resection of the cyst walls [18,19]. The best treatment for symptomatic patients appears to be total removal; however, radical resection may not always be feasible if the cyst is adherent to surrounding vital structures. For a favorable prognosis, complete excision of the cyst walls and its content is advised [9]. An MRI or CT scan must be performed on a long-term basis to check for the return of any residual lesion [14,20]. Comparisons of our case with other cases present in the literature are illustrated in Table 1. 

**Table 1:** Comparison of our case with other cases present in literature.

Patient with reference	Age(year) /Gender	Location of cyst	Malignant lesion/ Immunohistochemistry	Treatment	Outcome
Present Case	50 /F	Supratentorial	Adenocarcinoma. IHC: CK7, CK20, CDX2, TTF1, p53, with high MIB-1 labeling index	Gross total resection, Craniospinal Radiotherapy 50Gy	Death 11 months post op
Patient 1 [4]	Late 50’s/M	Right cerebral hemisphere	Mucinous adenocarcinoma IHC: CK5, MUC5AC, Ki-67>80%	Gross total resection	Stable
Patient 2 [5]	58/F	Right parietal lobe	Invasive mucinous papillary cystadenocarcinoma IHC: EMA, CAM5.2, CEA, CK19, CK7, CK20	Gross total resection	Stable
Patient 3 [7]	58/F	Left side of the foramen magnum	Well differentiated Papillary adenocarcinoma IHC: CEA, EMA, Ki-67= 20-40%	Subtotal resection	Sccumbed
Patient 4 [8]	46/M	Left cerebellopontine angle	Well differentiated adenocarcinoma. IHC: ki-67>20%	Partial resection, radiotherapy (50.4Gy)	Stable
Patient 5 [9]	26/F	Left cerebellopontine angle	Atypical appearance of were seen in some areas showing increased cellular density, relocation of the nuclei to the top of the cells, nuclear pleomorphism, mitosis and stromal invasion. IHC: EMA, CK, CA19- 9, CEA, Ki-67>50%	Gross total resection, radiotherapy (50.0Gy)	Dissemination
Patient 6 [10]	25/M	Right CPA extending toward cerebellar vermis	Mucinous low grade papillary adenocarcinoma arising in an EC. IHC: CK, EMA, CEA	Partial resection.	Recurred 4 months later, succumbed
Patient 7 [11]	45/F	Right parietal lobe	Papillary adenocarcinoma. IHC: EMA, CEA, CK	Gross total resection	Stable
Patient 8 [12]	53/M	Left anterior cervicomedullary junction	A typical neurenteric cyst with a one-layer epithelium in the first specimen to an adenocarcinoma with papillary proliferation in the second. IHC: EMA, CEA, CA19-9, CK7, CK-20, Ki-67=6.7%, p53=8% in the second specimen compared to 0% for both markers in the first specimen	Partial resection in the recurred tumor, radiotherapy (50Gy)	Recurred 3.5 years after total resection of the first tumor, succumbed one year after the second operation
Patient 9 [13]	36/M	Cisterna magna	Focally the epithelium showed branching papillary fronds to mould a cribriform, nuclear atypia with occasional pleomorphism, hyperchromasia, prominent nucleoli and clumping of chromatin, features of a dysplastic epithelium\intraepithelial carcinoma. IHC: AE1/AE3, Ki-67>80%	Gross total resection	Stable

From the past reports, it is evident that both males and females can be affected with no gender bias. In comparison with previously reported cases, our case is one of the few supratentorial lesions in which the lesion was localized to the velum interpositum and the trigone of the left lateral ventricle, a location uncommonly involved in NECs [4,5,11]. The majority of the cases are located in the posterior fossa and cerebellopontine angle [7-10,12,13].

Histologically, our case showed features of adenocarcinoma with immunoreactivity to CK7, CK20, CDX2, TTF1, CDX2, and p53, along with a high MIB-1 labeling index. This immunophenotype is to some extent similar to other cases with adenocarcinomatous changes [4,5,7-13]. The case under discussion showed CDX2 and TTF1, which is not common in others. This dual expression has not been previously reported in a single case. These findings suggest not just malignant potential but also the possibility of a broader differentiation lineage or molecular profile influencing tumor behavior and prognosis. The majority of the cases also demonstrated a high proliferation index with a percentage of 80% [4], 20-40% [7], >20% [8], >50% [9], 6.7% [12], and >80% [13]. To our knowledge, this is the first reported case in the literature in which CSF cytological analysis confirmed rare, atypical epithelial cells.

Surgical intervention varied among the reported cases, ranging from gross total to subtotal or partial resections, with or without radiotherapy. In comparison to other cases reported in the literature [4,5,7-13], our case uniquely presented with diffuse craniospinal leptomeningeal spread evident on MRI just months after the initial imaging, a feature not reported to this extent in previous cases. Similar findings were seen in the cases by Kimura et al., where recurrence and craniospinal spread were noted, and by Wang et al. [9]. This is in contrast to other cases, such as by Surash, which showed either localized recurrence or stable disease after resection and radiotherapy [8]. The dramatic clinical decline post-surgery despite GTR and craniospinal irradiation sets our case apart. This outcome parallels the case described by Sahara et al., where recurrence occurred within months despite multimodal therapy [12]. 

In general, malignant transformation of neurenteric cysts (NCs) within the central nervous system is extremely uncommon. Based on the findings of this case and previous analysis of the literature, a diagnostic criterion for identifying malignant NCs can be established. The anatomical distribution of NCs that undergo malignant transformation differs from that of typical NCs. Typically, these malignant transformations are seen in adults and are accompanied by unusual radiological features. Consequently, adult patients with either a primary or recurrent NC showing unusual radiological characteristics may have a higher risk of malignancy. The best course of treatment for NEC that has undergone malignant transformation is surgery, and close follow-up is necessary [7]. More research is needed to determine the therapeutic efficacy of adjuvant chemotherapy and radiation.

In summary, our case shares some similarities with previously reported cases in terms of age and histology but stands out due to its supratentorial location, early dissemination, and poor prognosis. The outcome emphasizes the unpredictable behavior of these lesions and the need for close monitoring, even after apparently complete resection. More comprehensive molecular studies and long-term follow-up data are essential to better understand prognostic indicators in malignant neurenteric cysts.

## Conclusions

This case emphasizes that NCs should be taken into account when making a differential diagnosis of cystic lesions of the central nervous system (CNS). Malignantly transformed NCs are difficult to diagnose preoperatively due to their non-specific clinical picture along with radiological features. In this context, the final diagnosis is made by histopathological examination and immunostaining of the surgically removed specimen. The presence of benign epithelium along with the transition to dysplastic epithelium with malignant features in histological examination, supported by immunohistochemistry studies including mainly the MIB-1 labeling index, which demonstrates a high proliferation index in the dysplastic epithelium compared to a low proliferation index in a benign epithelium, confirms the malignant transformation of the lesion.
